# Panel Doctors and Professional Cowardice

**Published:** 1914-05-30

**Authors:** E. H. Worth

**Affiliations:** 2 Aldrington Road, Streatham


*To the Editor of* The Hospital.

### Panel Doctors and Professional Cowardice.

*To the Editor of* The Hospital.

Sir,—It is all very well for Dr. Macnaughton Jones to advise us to leave those things that are behind and look forward to things that are to come, but the fact is that the profession has made such a pitiable spectacle of itself that I for one cannot get the taste of the thing out of my mouth.

Take, for instance, Colchester. The *Lancet's* account of the so-called fight in this week's paper almost makes one cry with anger, if one has any pride in the profession to which he belongs. The whole of the doctors in that important town let one—Worthington Evans, M.P.—frighten them by the whole-time bogey as if they were a set of second-rate thieves afraid of their own shadow, and as if their patients were going to leave them for some undressed bounders that Mr. Lloyd George or anyone else could send down.

Unless the panel doctors are brought to see their own cowardice, how can they be taught to fight like men, and how are we to get a good medical service if the last rout is to be forgotten and never mentioned?—Yours truly,

E. H. Worth, M.R.C.S.

2 Aldrington Road, Streatham, May 22, 1914.

